# Chlorophyll Supplementation Delays Aging in *Drosophila melanogaster* via Enhanced Stress Resistance and Detoxification Network Remodeling

**DOI:** 10.3390/nu18091465

**Published:** 2026-05-03

**Authors:** Suxia Shen, Ning Xu, Zhaotian Yang, Zixuan Han, Lin Zeng, Ajibola Nihmot Ibrahim, Yan Zhang

**Affiliations:** 1 College of Food Science and Nutritional Engineering, China Agricultural University, Beijing 100083, China; shen_sx573776028@163.com (S.S.); siriussalazar@163.com (N.X.); yangzhaotian1997@163.com (Z.Y.); better2802@163.com (Z.H.); zenglin6016@163.com (L.Z.); aomawunmi@gmail.com (A.N.I.); 2 Sanya Institute of China Agricultural University, Sanya 572025, China; 3 Key Laboratory of Fruits and Vegetables Processing, Ministry of Agriculture and Rural Affairs, Beijing 100083, China; 4 Frontier Technology Research Institute of China Agricultural University in Shenzhen, Shenzhen 518000, China

**Keywords:** chlorophyll, anti-aging, antioxidant, stress resistance, Nrf2, *Drosophila melanogaster*

## Abstract

Background: Chlorophyll (Chl), widespread in fruits and vegetables, has been shown to have numerous nutritional functions, including beneficial effects on obesity. However, whether Chl has an anti-aging effect remains unclear. Methods: Here, we studied the beneficial effects and mechanism of Chl on delaying aging using a *Drosophila* model. Results: The results showed that dietary supplementation of Chl in an appropriate dose (3.925 mg/L) significantly extended the lifespan (7.66–13.94%), improved climbing ability, increased CAT activity, reduced MDA content, enhanced stress resistance to starvation, heat stress, and cold shock in *Drosophila*. Notably, lifespan extension was not associated with dietary restriction, reproductive sacrifice, or circadian rhythm regulation. RNA-Seq analysis showed that Chl supplementation led to differential expression of 723 genes in female flies and 435 genes in male flies. KEGG analysis revealed that these differentially expressed genes were significantly enriched in the xenobiotic metabolism (XM) pathway. Within this pathway, phase II detoxifying enzyme genes associated with the CncC (Nrf2) signaling pathway (*GstD10*, *GstE7*, *Ugt37A3*, and *AOX2*) were significantly downregulated in both sexes. In contrast, protective target genes from the same pathway (*cat*, *Mrp4*, *Hsp68*) were significantly upregulated, as confirmed by qPCR. Conclusions: Taken together, our data indicate that Chl supplementation delays aging in *Drosophila melanogaster* via enhanced stress resistance and remodeling of the detoxification network.

## 1. Introduction

Aging in humans is a complex biological process characterized by a time-dependent progressive decline in physiological integrity, resulting in functional impairment and heightened susceptibility to mortality [1]. This degenerative process is associated with several hallmarks of biological alterations, including reduced food intake, decreased mobility, diminished reproductive capacity, impaired antioxidant defenses, and an imbalance in gut homeostasis [2]. These degenerative processes represent the foremost risk factor for major human pathologies, including cancer [3,4]. Therefore, promoting “healthy aging”—the maintenance of functional capacity in later life by mitigating the progression of age-related pathologies—has become a critical imperative for us [5,6].

Aging is not only a change in appearance but also involves the comprehensive effects of genes, cellular functions, metabolic disorders, and environmental factors [7,8]. The genetics of aging research has revealed a complex network of interacting intracellular signaling pathways and higher-order processes. Many of the pathways and processes, such as dietary restriction, that have been identified are known to be critical in homeostatic responses to environmental change [9]. Studies have established that numerous genes are implicated in their regulation. For example, activation of the single *forkhead box o* (FoxO), which is inhibited by phosphorylation via the InR/IRS/Akt axis, results in a robust lifespan extension in *Drosophila* [10]. These results offer novel insights into the genetic regulation of aging processes.

While altering the progression of aging at the organismal level remains highly challenging, achieving relative rejuvenation at the cellular or tissue level is feasible. This can be accomplished through the application of bioactive plant-derived compounds or advanced technological interventions [11,12]. Studies have found that certain phytochemicals, such as polyphenols, lycopene, aspirin, and berberine, can decelerate the aging process, extend lifespan, or mitigate aging-related diseases [13,14,15]. Some phytochemicals may induce hormesis, a biphasic dose–response phenomenon where low doses elicit stimulatory benefits while high doses exert inhibitory effects [16]. For instance, 200 μΜ Epigallocatechin-3-gallate (EGCG) significantly extended the lifespan of *C. elegans*, while 1000 μΜ EGCG reduced worm longevity [17]; similarly, Proshkina et al. [18] reported that short-term and intermittent quercetin exposure was more effective in lifespan extension than long-term continuous exposure in *D. melanogaster*. Such effects, usually driven by appropriate low doses, enhance brain resilience, improve organismal stress tolerance, and slow aging by activating host defense pathways (e.g., Nrf2 antioxidant signaling) and stress-tolerance genes (e.g., *GST*, *Hsp70*) [19,20,21]. Chlorophyll (Chl), the most abundant plant secondary metabolite in nature, with an annual production exceeding 1.2 billion tons [14,22], plays pivotal roles across multiple domains. Its applications span from food coloring and functional food additives to biomedical materials and renewable energy systems [23,24]. Additionally, emerging evidence from recent research reveals Chl’s bioactive potential in areas of obesity suppression, gut microbiota modulation, and lipid metabolism regulation [25,26,27]. However, there is almost no research on Chl’s anti-aging properties and the underlying mechanisms.

*Drosophila melanogaster* has a rich history in biological research, contributing to many key discoveries in genetics, development, and neurobiology. Thegenome contains~63% that have human orthologs [28]~75% of known human pathogenic genes are homologous to fruit flies [29,30]. The model organism is characterized by its small size, short lifespan, and rapid reproductive cycle, making it an ideal system for investigating the anti-aging mechanisms of natural extracts [11]. This study employs the *Drosophila* model system to investigate the anti-aging mechanisms underlying Chl’s age-delaying effects.

## 2. Materials and Methods

### 2.1. Chl Extraction and Purification

Chl extraction was performed using the method of Li et al. [31] Briefly, fresh spinach leaves of *Spinacia oleracea* ‘Super King’ (Japanese cultivar) harvested in May from Linyi City, Shandong Province, China, were homogenized using a T25 digital ULTRA-TURRAX^®^ homogenizer (IKA Works GmbH & Co. KG, Staufen, Germany) with a mixture of anhydrous ethanol and petroleum ether. The homogenate was centrifuged (CR21GII, Hitachi Group, Tokyo, Japan), and the supernatant was subjected to phase separation in a separatory funnel. The petroleum ether phase (containing Chl) was washed, dried, and then concentrated via rotary evaporation (RE-5210A, Shanghai Yarong Biochemical Instrument Factory, Shanghai, China) at 36 °C under reduced pressure. The purification was performed by column chromatography on neutral alumina (200–300 mesh) with stepwise elution, mainly removing carotenoids and lutein. Finally, the organic solvent in the purified Chl was removed using a multifunctional nitrogen blower, and Chl (Chl a:Chl b ≈ 3:1) [32] with a purity of 86.6% was obtained [33] and stored at −80 °C in the dark. The remaining minor components (13.4%) mainly consisted of homologous lipophilic pigments, Chl degradation products, and lipids.

### 2.2. Media Preparation, Drosophila Stocks, and Husbandry

Media preparation was performed according to the methodology of Dr. Juan Du’s laboratory (China Agricultural University, Beijing, China). The media used for larval development and longevity assays are shown in Table 1. For the yeast medium, combine water, CaCl_2_, sucrose, and dextrose and mix well. Increase heat and add cornmeal and yeast; boil for 10 min; add agar, and keep boiling for 5 min. Allow food to cool to 60 °C; add ethyl paraben and propionic acid; and dispense. The yeast was bought from Angel Yeast Co., Ltd. (low-sugar, high-activity dry yeast powder, Yichang City, Hubei Province, China). Chl groups were to add Chl to the yeast medium to a final concentration of 15.7 mg/L, 7.85 mg/L, and 3.925 mg/L, respectively, before it was dispensed. As Chl is easily degraded by light, the media prepared were stored in the refrigerator at 4 °C and protected from light, and were freshly prepared every six days. The Chl content in the culture medium was determined based on a literature review, with comprehensive consideration of daily vegetable intake, equivalent dose conversion, typical dosage ranges of plant extracts, and the solubility properties of chlorophyll, and was further confirmed through preliminary experiments.

Grape juice medium was used to collect *Drosophila* eggs. The preparation method is to boil pure water, sucrose, and agar in a pot. Add grape juice, stir over low heat, cool to 60 °C, add nipagin, evenly dispense, and store in the refrigerator at 4 °C, protected from light after cooling and solidification. When using it, apply a small amount of yeast paste (3 g of yeast and 10 g of water) on the surface of the medium.

The wild-type *Drosophila melanogaster* Canton-S and W^1118^ (NO.5905) stocks were obtained from Dr. Juan Du’s laboratory (China Agricultural University, Beijing, China). Fly stocks were maintained on standard food in an incubator at 25 °C, 60% humidity, with a 12 h light/12 h dark cycle unless otherwise stated. Flies for aging were collected and transferred to fresh food every 3 days.

Prior to all experiments, fly cultures were maintained at constant density for at least 2 generations. Embryo collections were synchronized to ensure developmental uniformity [34].

### 2.3. Survival Analysis

The lifespan assay was conducted as described by Libert et al. [34] and Castillo-Quan et al. [35] Adult flies were collected over 24 h and promptly transferred to yeast food. Following collection, flies were allowed to mature and mate for 48 h, after which males and females were separated under light CO_2_ anesthesia and placed in vials with appropriate food (Canton-S, *n* = 160 to 200 per group; W^1118^, *n* = 200 to 260 per group, 15 to 20 per vial). In subsequent experiments, unless otherwise specified, collection and processing were according to this procedure. Vials were stored on their side, which allowed the flies to wander away from the food even at an advanced age. Vials were changed every 3 days, at which time dead flies were removed and counted. Survival curves were tracked until all flies had died, and the mean, median, and maximum lifespans of the flies were determined.

There were five groups for each fly stock and each sex. One standard food group was maintained in an incubator with a 12 h light/12 h dark cycle as a daily reference group. The blank control group and three Chl experimental groups, as shown in Section 2.2 were maintained in an incubator under continuous 24 h dark conditions.

For starvation resistance, flies were collected as described and placed on yeast food to age for 10 days. Flies were then placed in fresh vials containing 1.5% agar with 3% ethyl paraben (10% *w*/*v*) and 0.5% propionic acid (*n* = 100 per group, 10 per vial). Vials were changed daily, and the number of dead flies was recorded every 12 h until all flies died, and survival curves were constructed.

### 2.4. Climbing Index Measurement

After collection and mating, as in the longevity assays, 15-day-old adult flies were placed in a vertical empty tube and adapted for 5 min (*n* = 80 to 100 per group, 10 per vial). After gently shaking the flies to the bottom of the tube, the flies habitually climbed upwards. The number of flies that reached the top of the tube within 10 s was recorded (A). Each group of flies was tested at least 5 times, with a minimum interval of 5 min between each test, and shaken with a similar force each time. The climbing index was calculated as CI = A/total.

### 2.5. Feeding and Body Weight Assays

Food intake detection was determined according to the method of Libert et al. [34]. After collection and mating as outlined for the longevity assays, 15-day-old adult flies were weighed and then fed standard food supplemented with 0.5% (*w*/*v*) FD&C Blue #1 (Shanghai Dye Research Institute Co., Ltd., Shanghai, China). FD&C Blue #1 (Synonyms: Acid Blue 9, Erioglaucine; Brilliant Blue FCF, C_37_H_34_N_2_O_9_S_3_Na_2_) was shown to be harmless to flies and is routinely used to color human food, drugs, and household chemicals. The dye does not decompose at low pH and is thermo-stable. Flies were exposed to dyed food for 20 min, after which groups of 5 female/male flies (8–10 groups per sample) were homogenized in 200 μL of PBS and cleared by centrifugation at 8000× *g* for 10 min. After centrifugation, the absorbance of the supernatant was measured at 625 nm.

### 2.6. Reproduction Assays

Flies were collected as virgins within 6 h of eclosion. Following collection, the virgins were placed on yeast medium and aged for 24 h. Then, males and females were separated and randomly assigned to groups. After 10 days of feeding with appropriate medium, ten replicate vials of 10 females and 15 males were established containing the grape juice medium (covered with yeast paste: 3 g yeast and 10 g water) for 24 h to stimulate egg production (10 vials per group). The flies were then removed, and egg counts were performed on day 5.

### 2.7. Circadian Rhythm Measurement

Circadian rhythm of individual male flies was measured using the *Drosophila* Activity Monitoring (DAM) System (DAM2, Trikinetics, Waltham, MA, USA) [36]. Male flies (aged 15 days) were loaded individually into glass tubes with a length of 65 mm and an inner diameter of 5 mm. The flies (*n* = 16) were entrained to a 12 h light/12 h dark cycle for 3 days and then released to constant darkness for at least six days to measure their rhythmicity. Chi-squared periodogram analysis determined whether a fly was significantly rhythmic (α = 0.05). Data analysis was done on a Macintosh computer running the FaasX (Fly activity analysis suite, 1.22 (32-bits)) software (obtained from https://trikinetics.com, NeuroPSI, France). Individual flies with power (≥10) and “width” values of 1.5 or more were considered rhythmic [37]. Activity data were collected using MATLAB (R2022a, MathWorks, Natick, MA, USA) for each fly, and were binned every 30 min for the circadian rhythm analyses.

### 2.8. Hydrogen Peroxide (H_2_O_2_) Challenge Test

For the acute oxidative stress test, this study employed a modified hydrogen peroxide (H_2_O_2_) challenge assay, based on the method of Wongchum et al. [38] Hydrogen peroxide was diluted with 6% glucose solution to a final concentration of 30%, and 200 μL of the diluent was added onto a filter paper. Flies were then transferred to measuring tubes lined with the H_2_O_2_ filter paper, with 10 flies per tube and 10 replicate tubes per group. The number of fly deaths was counted every 3 h until all were dead, and a survival curve was drawn.

### 2.9. Antioxidant Enzyme Activities and MDA Content

A sample (more than 100 flies per sample) of males or females of 15-day-old adult flies that were fed base and test diets was collected, respectively. Fruit flies were anesthetized with CO_2_, and a tissue homogenate was prepared by adding physiological saline (5 flies with 100 μL). The homogenate was centrifuged in a low-temperature centrifuge for 15 min (4 °C, 8000 r/min). Malondialdehyde (MDA) levels, superoxide dismutase (SOD) activity, and catalase (CAT) were measured separately by using MDA kit (cat. BC0025), SOD kit (cat. BC0175), and CAT kit (cat. BC0205) produced by Beijing Solarbio Science & Technology Co., Ltd. (Beijing, China).

### 2.10. Environmental Stress Test

Fifteen-day-old adult flies (more than 100 flies per sample, 10 flies per vial, 10 vials per sample) were prepared for testing. In the cold shock recovery experiment, we exposed all flies to a 4 °C environment until their complete activity ceased. Afterward, the fruit flies were transferred to room temperature, and the time required for recovery from cold shock was recorded. For the heat stress resistance experiment, the flies were kept in a constant temperature incubator at 39 °C, and their survival status was recorded every hour until they all died. Survival curves were drawn at the end.

### 2.11. Triglyceride and Glucose Assays

After collection and mating as outlined for the longevity assays, 15-day-old adult flies (10 females or males) were transferred into a 1.5 mL centrifuge tube and quick-frozen in liquid nitrogen (10 tubes per group). After homogenization in 200 μL of 0.05% PBS/Triton-X buffer, the triglyceride content of each group was determined according to the TAG kit method (# A110-2-1, Nanjing Jiancheng Bioengineering Research Institute Co., Ltd., Nanjing, China). For the glucose assay, the flies were starved for 1 h, centrifuged, and the supernatant was taken. The glucose was then determined using the glucose content detection kit method (Beijing Solarbio Science & Technology Co., Ltd., Beijing, China) according to the instruction manual.

### 2.12. RNA Sequencing

RNA sequencing was performed as described by Zhu et al. [2]. Briefly, the female or male flies (Canton-S) were cultured in the standard medium and in medium supplemented with 3.925 mg/mL Chl for 15 days. Total RNA was extracted from different groups, and the integrity was determined by the Bioanalyzer 2100 system (Agilent Technologies, Santa Clara, CA, USA). The total amount of RNA samples was detected by the ND-2000 (Thermo Fisher Scientific, Waltham, MA, USA). Then, mRNA was enriched from total RNA and randomly fragmented. After library construction and quality inspection, transcriptome sequencing was performed on the Illumina platform (Illumina, San Diego, CA, USA). To identify DEGs (differentially expressed genes), the expression level of each transcript was calculated according to the transcripts per million reads (TPM) method. Subsequently, DESeq2 (v1.49.4, Bioconductor, USA & Germany) was used for differential expression analysis, and the ClusterProfiler R package (v4.0.2, YuLab-SMU, Southern Medical University, Guangzhou, China) was used for GO and KEGG pathway enrichment analysis. RNA-seq was performed at Majorbio Bio-pharm Biotechnology Co., Ltd. (Shanghai, China). Finally, based on the differentially expressed transcription factor (TF) genes shared between males and females annotated from RNA-seq data, upstream regulators of these differentially expressed TF genes were predicted, and their motif analyses were performed using RStudio (version 2026.01.2-418, Posit PBC, Boston, MA, USA) combined with the AnimalTFDB and JASPAR databases (1000 bp–0 bp, Cor > 0.5).

### 2.13. Quantitative RT–PCR (qRT-PCR)

Total RNA was extracted from approximately 10 flies using an extraction reagent kit (R1200, Beijing Solarbio Science & Technology Co., Ltd., Beijing, China). The cDNAs were synthesized by reverse transcription using HiScript III RT SuperMix for qPCR (+gDNA wiper) (R323, Nanjing Vazyme Biotech Co., Ltd., Nanjing, China). The cDNA preparation was then subjected to real-time quantitative PCR (SYBR Green I Chimeric Fluorescence Method) according to the protocol of Taq Pro Universal SYBR qPCR Master Mix (Q712, Nanjing Vazyme Biotech Co., Ltd., Nanjing, China). 2^−ΔΔCT^ method was used to calculate the relative expression of mRNA of each gene. All the primers used are listed in Table 2.

### 2.14. Statistical Analysis

Each experiment was performed at least in triplicate. The results obtained were analyzed and the graphs were drawn using Prism 9 (Version No.6, GraphPad, San Diego, CA, USA). Data are presented as the mean ± standard deviation (S.D). Comparisons of survival rates between groups were conducted using the log-rank test for significance. Apart from survival rates, one-way analysis of variance (ANOVA), *t*-test, and chi-square test were used to determine statistical significance. The differences between groups were considered statistically significant at *p* < 0.05 for all tests, and significance was denoted as * *p* < 0.05, ** *p* < 0.01, *** *p* < 0.001, and **** *p* < 0.0001.

## 3. Results and Discussion

### 3.1. Effects of Chl Supplementation on the Lifespan and Athletic Ability in Drosophila

As age increases, the body’s resistance to harsh external environments decreases, causing some aging-related diseases and affecting life expectancy [39]. Studies have shown that lifespan is the most direct reflection of aging [40], and motor ability is negatively correlated with aging. Therefore, we detected the effect of Chl supplementation on the healthspan of fruit flies through lifespan and climbing ability.

**Figure 1:**
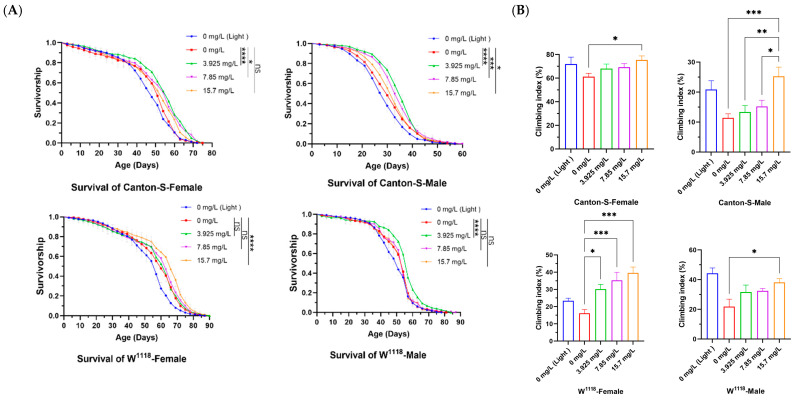
Effects of Chl on the lifespan and athletic ability of *Drosophila*. (**A**) Survivorship curves; (**B**) Climbing ability. Data are presented as the mean ± SD. In panel (**A**), the *p*-values were determined by log-rank test. In panel (**B**), the *p*-values were determined by one-way ANOVA with Dunnett’s multiple comparisons test. * *p* < 0.05; ** *p* < 0.01; *** *p* < 0.001; **** *p* < 0.0001 vs. the control group. ns, no significant difference between the two groups.

Female and male *Drosophila* were reared on media containing different concentrations of Chl for the lifespan test. Compared with the control group, Chl supplementation at a certain concentration significantly increased the survival rate, mean lifespan, median lifespan, and maximum lifespan in flies (Figure 1A and Table 3). For the Canton-S (female and male) and W^1118^ (male), 3.925 mg/L was the optimal concentration for addition; while, for W^1118^ (female), the optimal concentration was 15.7 mg/L. Among them, the mean lifespan increased by 12.01% (from 46.71 to 52.32 days, *p* < 0.001), 13.93% (from 30.79 to 35.08 days, *p* < 0.0001), 8.01% (from 56.33 to 60.84 days, *p* < 0.001), and 7.66% (from 50.42 to 54.28 days, *p* < 0.0001), respectively. This suggested that there is an optimal dose of Chl for lifespan extension, and Chl has a greater impact on the lifespan of Canton-S than W^1118^. It is worth noting that the Chl concentration producing the optimal anti-aging effect was not the highest, but a relatively moderate dose among the experimental groups. This is consistent with hormesis to some extent, whereby nutrients or natural supplements exert anti-aging effects at relatively low doses [16]. These findings are also in line with several recent studies [41,42]. The results indicate that Chl supplementation can delay aging in *Drosophila*, but there are differences in the effects across varieties and genders.

A prior study, which focused on the oxidative stress tolerance of *Caenorhabditis elegans* (*C. elegans*) fed with Chl, demonstrated that Chl extends the lifespan of *C. elegans* by up to 25% [43]. This finding aligns with the results of our current research. Interestingly, increasing evidence shows that many food-derived small molecules, also called phytochemicals, can extend lifespan in various animal species, such as plant polyphenolics from strawberry, spinach, blueberry, and tea [13,44]. These studies also indirectly confirm that Chl prolongs the lifespan of fruit flies. In addition, many studies have found differences in experimental results among fruit fly varieties and gender [28,45]. Hodge et al. found that under unrestricted diet conditions, the lifespan of W^1118^ (white eye) is significantly prolonged, while the lifespan of Canton-S (red eye) remains unchanged in continuous darkness. The core reason for this difference may be that the white gene defect in W^1118^ leads to a lack of red pigment protection in its eyes, making it more sensitive to light damage. Therefore, the dark environment has a more significant effect on extending its lifespan [46].

As the body ages, physiological functions and self-regulation abilities gradually decline, and the crawling ability of fruit flies is an excellent indicator of the degree of aging and health status of the body. The better the ability to crawl vertically upwards against gravity, the younger the body state of the fruit flies [47]. The locomotor abilities of flies fed with or without Chl for 15 days were detected. Compared with control flies, the climbing ability of all flies fed with Chl improved, especially with 15.7 mg/L (Figure 1B). The results show that Chl supplementation could improve the motor behavior of flies and contribute to their health span.

### 3.2. Effects of Chl Supplementation on the Food Intake and Fecundity of Drosophila

Caloric restriction (CR) and dietary restriction (DR) have been shown to prolong the lifespan of multiple species [48]. Pharmacological intervention may extend lifespan via compensatory effects from the sacrificed reproductive capacity and reduced food intake that induces dietary restriction [34]. To eliminate interference from these factors, we tested whether Chl-fed flies exhibit altered feeding behavior or reproductive investment, which might explain their longevity effect.

**Figure 2:**
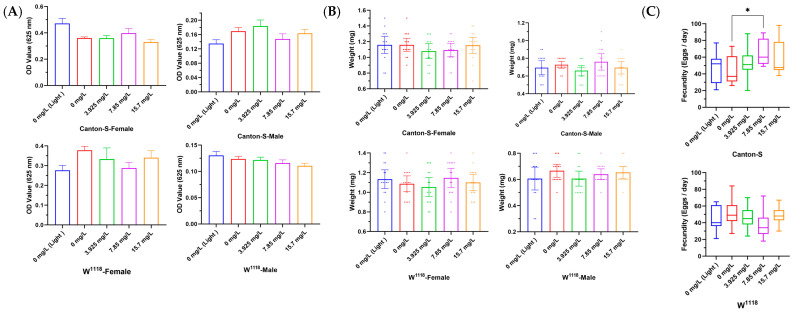
Effects of Chl on the (**A**) food intake (relative food consumption), (**B**) weight and (**C**) fecundity of *Drosophila*. All data are presented as the mean ± SD. The *p*-values were determined by one-way ANOVA with Dunnett’s multiple comparisons test. * *p* < 0.05 vs. the control group.

The results show that the food consumption of flies with Chl at three concentrations (3.925, 7.85, and 15.7 mg/L) was not significantly different from that of the control group (Figure 2A), suggesting that Chl did not alter the food intake behavior of flies. To confirm that Chl supplementation would not affect the food intake of flies, we analyzed the body weight of flies at 15 days after Chl addition. The results showed no obvious change in body weight of Chl-supplemented flies compared with the control group (Figure 2B), demonstrating that the extension of flies’ lifespan was not driven by additional calorie restriction.

In our experiments, the fecundity of treated flies was also not affected by Chl (Figure 2C). Interestingly, the reproductive rate of Canton-S fed with 7.85 mg/L Chl actually increased (from 43.27 to 66.67 eggs/day, *p* < 0.05). This may suggest that the regulation of chlorophyll in fruit flies is a “positive synergy” rather than a “trade-off” (most longevity interventions often accompany a decrease in reproductive capacity). In summary, the results indicate that Chl prolongs the lifespan of fruit flies without restricting dietary intake or sacrificing their reproductive capacity.

### 3.3. Regulatory Effect of Chl on the Circadian Rhythm in Drosophila

The circadian rhythm of fruit flies is primarily regulated by light signals, and prolonged darkness can gradually uncover and potentially alter their endogenous rhythms [49]. Research has indicated that there is a mutual interaction between circadian rhythm and aging [50]. To explore whether chlorophyll extends fruit fly lifespan by interfering with their circadian rhythm changes under dark conditions, we measured the circadian rhythm of 15-day-old male flies.

**Figure 3:**
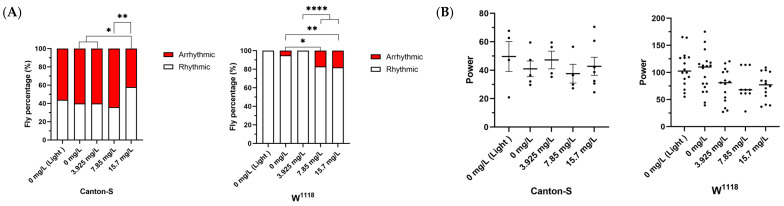
Regulatory effect of Chl on the circadian rhythm of *Drosophila*. (**A**) Percentage of rhythmic and arrhythmic flies; (**B**) Circadian power value of flies with rhythm. Data are presented as the mean ± SD. The *p*-values were determined by one-way ANOVA with Dunnett’s multiple comparisons test. * *p* < 0.05; ** *p* < 0.01; **** *p* < 0.0001.

It was found that the percentage of rhythmic flies of Canton-S was significantly improved from 40% to 58.33% when fed with 15.7 mg/L Chl (*p* < 0.05), indicating that Chl may interfere with the circadian rhythm of fruit flies. However, the W^1118^ strain showed opposite results under Chl treatment (Figure 3 and Table 4). We speculate that the regulatory effect on rhythm may be related to the concentration of Chl, and the Chl concentration administered to W^1118^ was not optimal. However, the circadian rhythm of fruit flies in the group of 3.925 mg/L Chl was improved to 100%, albeit not significantly. In addition, it was discovered that the circadian rhythm of W^1118^ was significantly stronger than that of the Canton-S group. The reason for this difference may be that Canton-S maintains its rhythm by receiving light signals through red eye pigments, and under dark conditions, its own rhythmicity is poor [46]. Therefore, Chl exerts a regulatory effect on the circadian rhythm of fruit flies, but this effect is relatively weak and lacks consistency with the lifespan experiment results. Therefore, intervening in fruit fly lifespan via rhythm regulation is not the primary pathway of chlorophyll.

### 3.4. Effect of Chl on the Oxidative Stress Resistance

**Figure 4:**
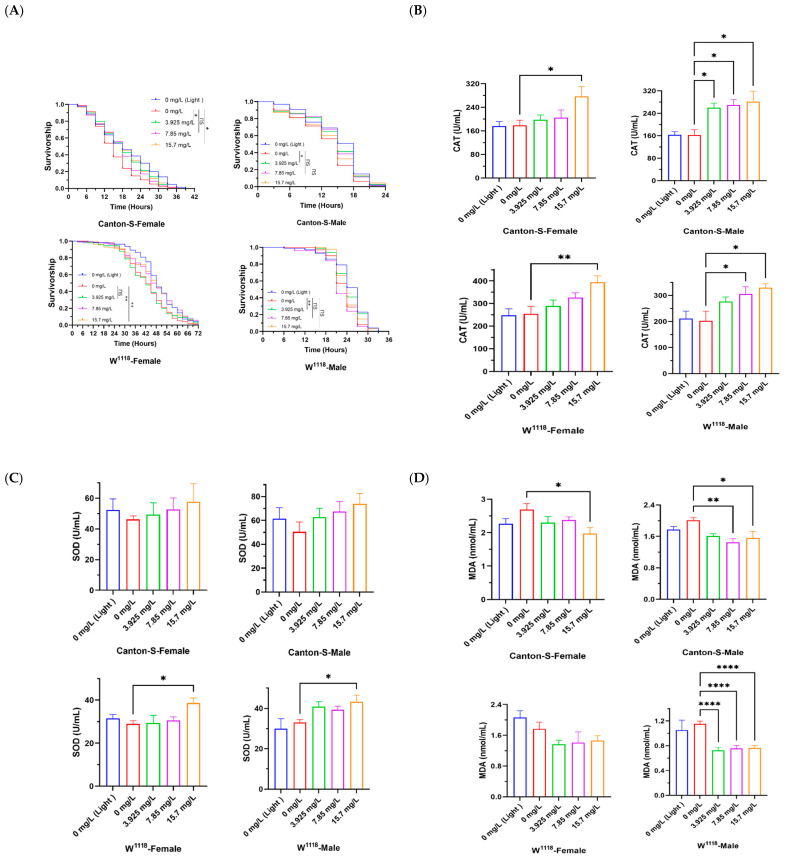

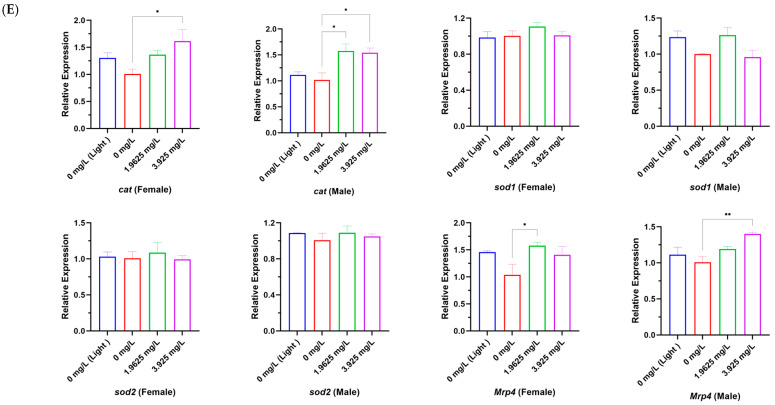
Effects of Chl supplementation on the antioxidant capacity in *Drosophila*. (**A**) Survivorship curves of flies induced by H_2_O_2_; (**B**,**C**) Activities of CAT and SOD; (**D**) The content of MDA; (**E**) qPCR analysis of antioxidant and detoxification-related genes. Data are presented as the mean ± SD. In panel (**A**), the *p*-values were determined by log-rank test. In panels (**B**–**D**), the *p*-values were determined by one-way ANOVA with Dunnett’s multiple comparisons test. * *p* < 0.05; ** *p* < 0.01 and **** *p* < 0.0001 vs. the control group. ns, no significant difference between the two groups.

The free-radical theory of aging postulates that oxidative damage underlies the aging process. Notably, a common feature of many lifespan-extending compounds is their potent antioxidant capacity [51]. H_2_O_2_ was employed to generate hydroxyl radicals (OH), which elevated reactive oxygen species (ROS) levels and accelerated aging in fruit flies upon induction [52]. This model was used to examine whether Chl extends lifespan by enhancing antioxidant capacity [2]. The results showed that supplementation with a specific concentration of Chl significantly improved the survival rate of flies under H_2_O_2_-induced oxidative stress (Figure 4A). In Canton-S flies, antioxidant capacity was significantly enhanced in both females and males treated with 3.925 mg/L Chl, the mean survival time of flies was extended by 17.6% (from 15.38 to 18.08 h, *p* < 0.05) and 11.1% (from 12.90 to 14.33 h, *p* < 0.05). In W^1118^ flies, males exhibited antioxidant capacity in the 3.925 mg/L Chl group, with mean survival time increased from 23.43 to 24.84 h (6%, *p* < 0.01); while females showed elevated antioxidant capacity in the 7.85 mg/L and 15.7 mg/L Chl groups, with stronger stress resistance observed in females at 15.7 mg/L Chl (mean survival time increased by 9.6%, *p* < 0.01). Interestingly, the antioxidant capacity across different groups followed a trend consistent with the earlier lifespan assay, and the antioxidant activity of the female group was greater than that of the male group. Together, these findings indicate that Chl can enhance the antioxidant defense system in fruit flies.

Furthermore, to investigate whether Chl extends the lifespan of *Drosophila* by enhancing antioxidant enzyme activity, the levels of superoxide dismutase (SOD) and catalase (CAT) and the antioxidant/detoxification-related genes (*cat*, *sod1*, *sod2*, and *Mrp4*) in Chl-treated fruit flies were investigated. As shown in Figure 4B,C, CAT activity was significantly increased in both Canton-S and W^1118^ strains in a dose-dependent manner following Chl supplementation. In the 15.7 mg/L Chl group, the CAT activity was increased by 54.8% (Canton-S, female, *p* < 0.05), 73% (Canton-S, male, *p* < 0.05), 55.7% (W^1118^, female, *p* < 0.01), and 62.9% (W^1118^, male, *p* < 0.05), respectively. While SOD activity was markedly elevated only in W^1118^ flies. Also in the 15.7 mg/L Chl group, the SOD activity was increased by 33.1% (W^1118^, female, *p* < 0.05) and 30.5% (W^1118^, male, *p* < 0.05). It can be seen that the effect of Chl on CAT activity in flies is greater than that on SOD. Malondialdehyde (MDA), a marker of lipid peroxidation, reflects the extent of oxidative damage in vivo. As illustrated in Figure 4D, MDA levels were significantly reduced upon Chl treatment in all groups except female W^1118^ flies. MDA levels were reduced by 26.5% (Canton-S, female, *p* < 0.05), 28% (Canton-S, male, *p* < 0.01) and 37.3% (W^1118^, male, *p* < 0.0001), respectively. The qPCR results were consistent with these findings (Figure 4E). In the Chl-treated groups at appropriate concentrations (3.925 mg/mL or 1.9625 mg/mL), the expression levels of *cat* and *Mrp4* genes were higher than those in the control group in both male and female *Drosophila*. However, the expression levels of *sod1* and *sod2* genes in Chl groups showed no significant changes compared with the control groups. Together, the change in the antioxidant capacity of fruit flies may be caused by Chl’s enhancement of the activities of antioxidant enzymes (mainly CAT) and its inhibition of the production of MDA.

These results suggest that Chl enhances the antioxidant capacity in *Drosophila* (in vivo), which is consistent with previous findings that reported that Chl significantly reduced the damage from juglone-induced acute oxidative stress, especially at low concentrations [43]. A similar finding has been reported for other phytochemicals, like resveratrol and quercetin, which could improve the ability to resist oxidative stress and prolong life by scavenging ROS [12,15,53]. Another study has suggested that the genetic pathways underpinning lifespan extension and stress resistance are likely interconnected [54]. The Nrf2 signaling pathway (the CncC analog) [55] is one such critical pathway. Furthermore, functional nutrients maintain cellular redox homeostasis by activating the core transcription factor Nrf2 (CncC), as well as its downstream stress-resistance genes (e.g., heat shock protein 68 and 70, *Hsp68* and *Hsp70*), antioxidant enzymes including superoxide dismutase (SOD), catalase (CAT), and glutathione S-transferase (GST), and related proteins such as the transporter Mrp4. This mitigates aging-associated oxidative stress and metabolic dysfunction, thereby delaying senescence and age-related disorders [56,57,58]. Given that Chl is a nutrient likely to exert hormetic effects, and Chl supplementation modulates the expression of *cat* and *Mrp4*, genes associated with the CncC (Nrf2) pathway, elevates catalase (CAT) activity in a dose-dependent manner, and reduces malondialdehyde (MDA) levels. These findings therefore indicate that Chl enhances antioxidant responses in *Drosophila* by regulating the CncC (Nrf2) signaling pathway, thereby exerting protective effects and extending lifespan. However, Chl does not activate the CncC (Nrf2) pathway canonically, as the mRNA expression levels of *sod1* and *sod2* showed no significant differences between the control and treatment groups, and SOD activity was also largely unchanged in the Canton-S strain.

Some studies have shown that antioxidant activity alone cannot fully explain longevity [59,60]. Therefore, the life-extending effect of Chl is likely not based solely on mitigating oxidative stress.

### 3.5. Effects of Chl on the Resistance to Starvation Stress, Heat Shock, and Cold Shock

Interventions that extend lifespan are often associated with enhanced stress resistance across multiple model organisms, and the CncC (Nrf2) pathway is also involved in regulating stress resistance in *Drosophila*. To determine if Chl delays aging by enhancing stress resilience in *Drosophila*, multiple physiological markers in 15-day-old flies were assessed. In addition to measuring the antioxidant capacity, their resistance to starvation, heat stress, and cold shock were evaluated.

**Figure 5:**
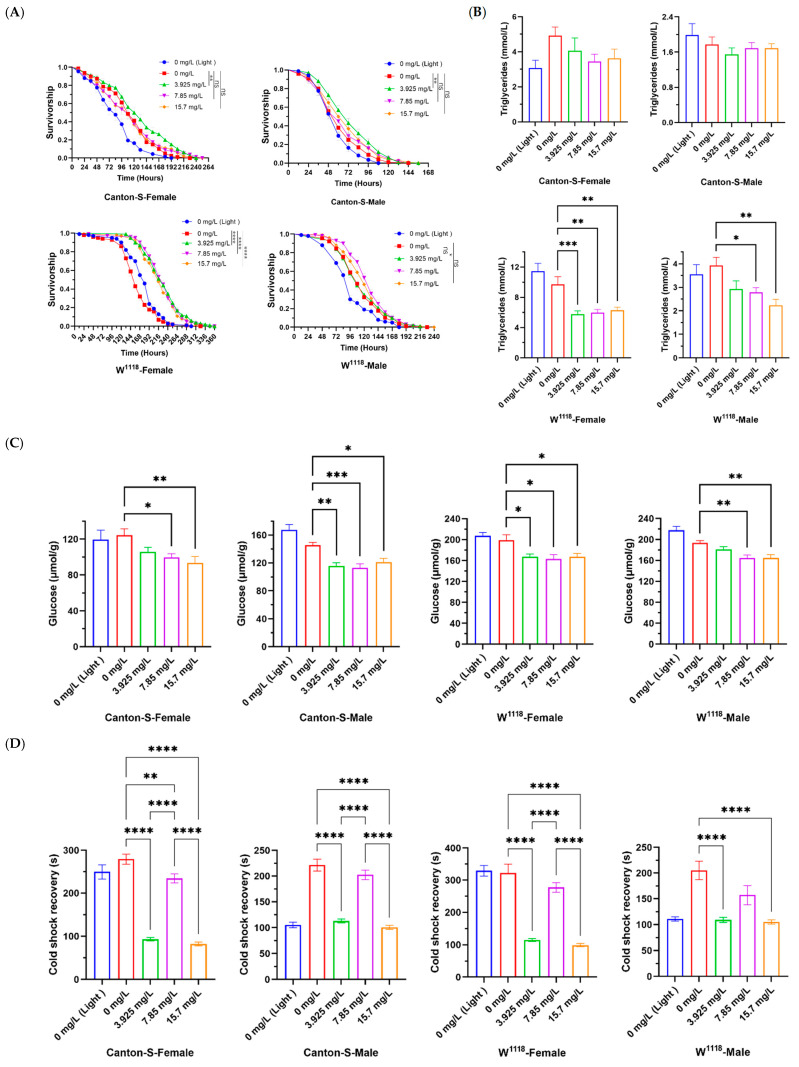

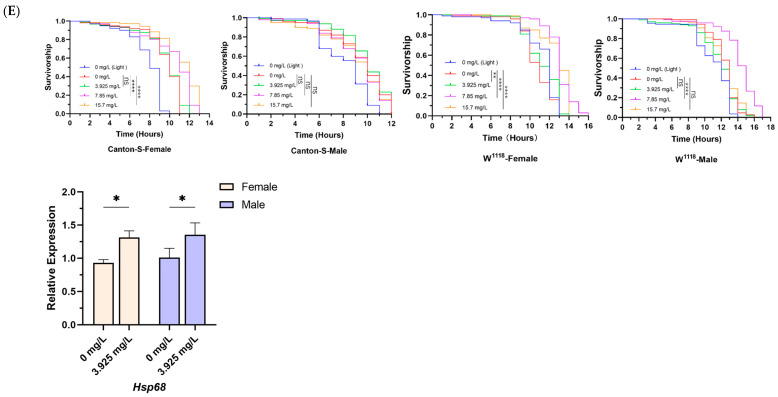
Effect of Chl on stress resistance in *Drosophila*. (**A**) Resistance to starvation; (**B**) The relative level of triglyceride; (**C**) The relative level of glucose; (**D**) Resistance to cold shock; (**E**) Resistance to heat stress and qPCR analysis of stress resistance-related gene. Data are presented as the mean ± SD. In panel (**A**,**E**), the *p* values were determined by log-rank test. In panels (**B**–**D**), the *p* values were determined by one-way ANOVA with Dunnett’s multiple comparisons test. * *p* < 0.05; ** *p* < 0.01; *** *p* < 0.001 and **** *p* < 0.0001. ns, no significant difference between the two groups.

Under starvation, fruit flies enter a state of stress and greatly shorten their lifespan [61]. We put *Drosophila* on an agar-only medium to provide water without nutrients, and monitored and recorded their deaths. As shown in Figure 5A, compared with the control group, 3.925 mg/L Chl extract extended the mean lifespan of Canton-S flies by 15.2% (from 111.6 to 128.6 h, female, *p* < 0.01) and 21.8% (62.16 to 75.72 h, male, *p* < 0.01), respectively. For W^1118^ flies, all Chl groups extended the mean lifespan of female flies (increased by 39.8% on average, *p* < 0.0001), and 7.85 mg/L Chl extended the mean lifespan of male flies (12.8%, from 114.7 to 129.4 h, *p* < 0.05). Simultaneously, it was observed that triglyceride (in W^1118^) and, more notably, glucose levels (in both Canton-S and W^1118^) were significantly reduced following chlorophyll intervention compared to the control (Figure 5B,C). These results suggest that Chl may contribute to the extension of *Drosophila* lifespan by affecting energy storage, and this may be related to its ability to inhibit α-amylase and α-glucosidase activities [27].

At 4 °C, *Drosophila* falls into a transient dormant state, and its body performance is greatly impaired [61]. The cold shock test was to judge whether the Chl has a slowing effect on the flies when they deal with the low-temperature, uncomfortable environment (Figure 5D). Compared with the control group, the flies in the 3.925 mg/L and 15.7 mg/L Chl groups woke up quickly and significantly (*p* < 0.0001). Resuscitation times were 93 s and 82 s (female, Canton-S), 113 s and 101 s (male, Canton-S), 115 s and 99 s (female, W^1118^), 109 s and 105 s (male, W^1118^), respectively. At the same time, the ability of Chl to protect *Drosophila* lifespan under high-temperature conditions was explored. As shown in Figure 5E, Chl can significantly prolong the lifespan of flies under heat stress (*p* < 0.0001), except in female Canton-S flies. After feeding with Chl, the mean lifespan of male and female W^1118^ flies was extended by 21.3% and 15.3%, respectively, and the mean lifespan of female Canton-S flies was extended by 16.4%, compared with the control group. Meanwhile, our results revealed that the expression level of *Hsp68* was also significantly upregulated, showing a positive correlation with the improved stress resistance in *Drosophila*. As a canonical direct downstream target gene of the CncC (Nrf2) pathway, *Hsp68* further supports that chlorophyll may enhance stress resistance and extend lifespan by modulating the CncC (Nrf2) signaling pathway. To sum up, flies fed with Chl show resistance to starvation stress, heat shock, cold shock, and upregulate the expression of *Hsp68*, suggesting that Chl may modulate the CncC (Nrf2) signaling pathway and enhance the overall health status and survival resilience of organisms.

### 3.6. Effect of Chl Supplementation on Gene Expression in Drosophila

**Figure 6:**
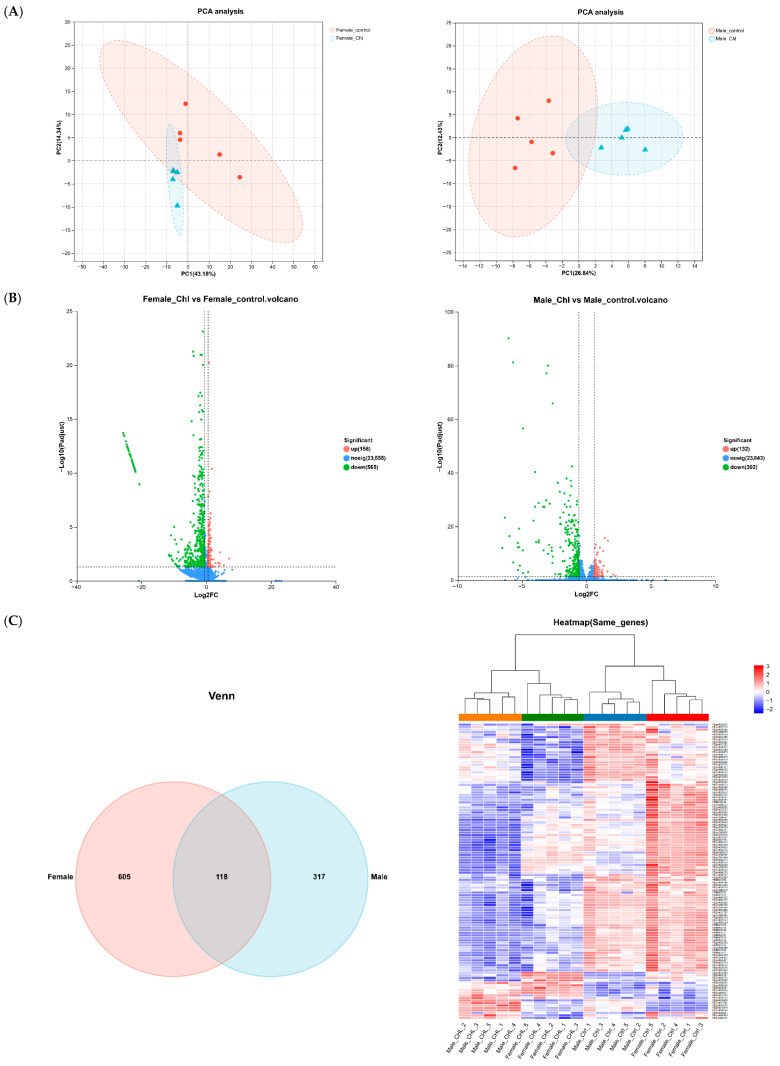

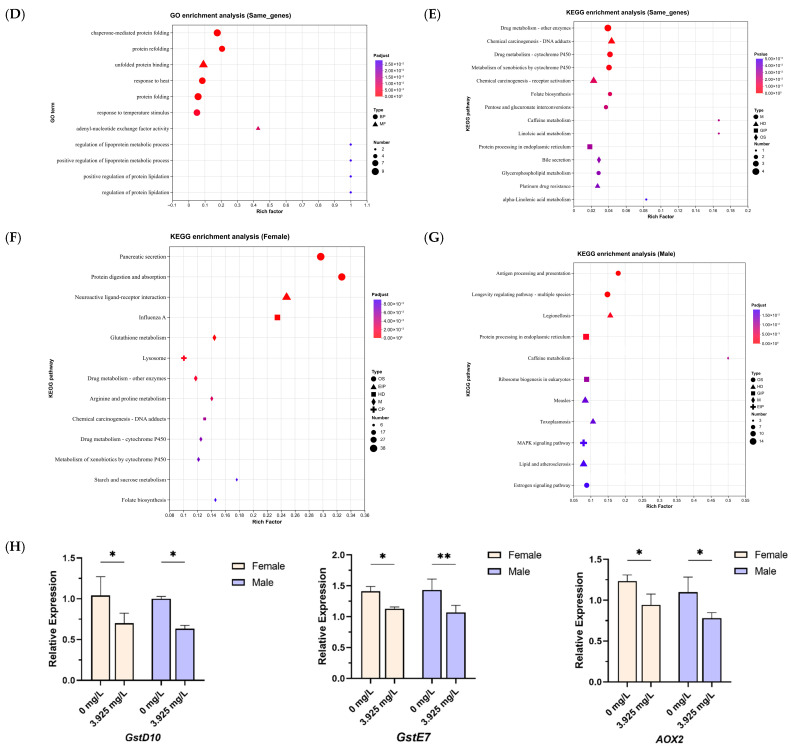
Effects of Chl supplementation on gene expression in *Drosophila*. (**A**) PCA analysis showing the similarity between samples; (**B**) Volcano plots showing the number of differentially expressed genes in flies fed with or without Chl (*p*-adjust < 0.05, FC ≥ 1.5); (**C**) Venn diagram and heatmap (same genes) showing the same differentially expressed genes between female and male group; (**D**) Gene ontology (GO) and (**E**) KEGG pathway analysis of the same differentially expressed genes between female and male group; (**F**,**G**) KEGG pathway analysis of the differentially expressed genes in female or male flies. (**H**) mRNA expression levels of the cofactors biosynthesis pathway-related genes. The type of GO analysis: BP, biological process; MF, molecular function. The type of KEGG analysis: M, metabolism; HD, human diseases; GIP, genetic information processing; OS, organismal systems; EIP, environmental information processing; CP, cellular processes. In panel (**E**), data are presented as the mean ± SD. The *p* values were determined by *t*-test. * *p* < 0.05 and ** *p* < 0.01 vs. the control group.

To comprehensively investigate the anti-aging mechanisms of Chl, RNA-sequencing was performed on the 15-day-old Canton-S fruit flies treated with or without Chl (control). The Canton-S strain was selected because both males and females exhibited stable and consistent responses to Chl at a concentration of 3.925 mg/L in lifespan, oxidative stress, and stress-resistance experiments. Therefore, this concentration was applied to the Chl treatment group. The expression profiles of approximately 20,000 genes were obtained from the sequencing database. Principal component analysis (PCA) revealed clear separation between sample groups and good reproducibility within groups (Figure 6A). Moreover, differential expression analysis was performed using the DESeq2 software (v1.49.4, Bioconductor, USA & Germany), and the screening conditions were set at *p*-adjust < 0.05 and |FC| ≥ 1.5. Compared to the control groups, long-term Chl treatment significantly altered the transcript levels of 723 genes in females and 435 genes in males. Specifically, in females, 158 genes were upregulated, and 565 were downregulated, while in males, 132 genes were upregulated, and 303 were downregulated (Figure 6B). The bioinformatic analysis revealed 118 same genes were regulated in both female and male groups (Figure 6C).

Gene ontology (GO) enrichment analysis demonstrated that the same genes affected by Chl were mainly involved in biological processes (BP), such as chaperone-mediated protein folding, protein refolding, response to heat, protein folding, and response to temperature stimulus. In terms of molecular function (MF), the most abundant GO functions were unfolded protein binding (Figure 6D). Owing to this, the genes affected by Chl are primarily involved in protein synthesis and modification, as well as in responses to environmental stimuli. Kyoto Encyclopedia of Genes and Genomes (KEGG) pathway enrichment analysis of differentially expressed same genes revealed that the following three signaling pathways were involved in the aging process. These pathways include drug metabolism-other enzymes, drug metabolism-cytochrome P450, and metabolism of xenobiotics by cytochrome P450 (Figure 6E). The result showed that the xenobiotics metabolism (XM) pathway exhibited a strong association with aging. Therefore, real-time PCR was used to verify gene expression levels in the XM pathway. The results also showed that, compared with the group without Chl treatment, the expression levels of these genes, including *GstD10*, *GstE7*, and *AOX2*, were significantly decreased after Chl supplementation in *Drosophila* (Figure 6H). The results suggest that the XM pathway indeed plays a crucial role in delaying aging by Chl in fruit flies.

The *GstD10* and *GstE7* are glutathione *S*-transferases (GSTs), a phase II antioxidant enzyme, that represent a major group of detoxification enzymes [62], catalyzing the nucleophilic attack of glutathione (GSH) on toxic electrophilic substrates and producing a less dangerous compound [63,64]. The GST genes are upregulated in response to oxidative stress and are inexplicably overexpressed in many tumors, leading to problems during cancer chemotherapy [64,65]. However, we found that although Chl supplementation significantly decreased MDA, a marker of oxidative damage, and increased CAT activities, it unexpectedly decreased the enzymatic activities of *GstD10 and GstE7*. This is similar to studies on some phytochemicals, such as quercetin, which have anti-aging activity [12,13], but also inhibits GST [66]. The responses of GST genes may protect the steady-state response of the organism [67]. In a study on *Hyphantria cunea* and their response to the oxidative stress caused by the infection of *Hyphantria cunea* nucleopolyhedrovirus (HcNPV), the expression levels of most GSTs were downregulated at low concentrations of HcNPV, and the reason may be that ROS levels were under the threshold of the body system [68]. Similarly, Chl and its metabolites possess potent direct antioxidant and electrophilic substance scavenging capacities [26], accompanied by a significant decrease in malondialdehyde (MDA) levels. Thus, our findings indicate that Chl also reduces the demand for GST-dependent detoxification pathways in *Drosophila*, thereby downregulating their activities through feedback to conserve resources and energy, and maintain organismal homeostasis.

Combined with the previous studies on *Drosophila* stress resistance (Section 3.4 and Section 3.5), the significantly differentially expressed genes can be roughly divided into two categories: one consists of canonical targets of CncC (Nrf2), which are associated with basic antioxidant defense, protein homeostasis, and toxin efflux, and belong to protective genes (*cat*, *Mrp4*, *Hsp68*; upregulated); the other is highly sensitive to intracellular ROS/electrophile levels and classified as stress-inducible (*GstD10*, *GstE7*, *AOX2*, *Ugt37A3*; downregulated). Therefore, the results of this study indicate that Chl does not activate the CncC (Nrf2) pathway canonically, but rather activates it moderately and selectively, upregulating protective target genes (*cat*, *Mrp4*, *Hsp68*). Meanwhile, the effective scavenging of ROS by Chl leads to the feedback downregulation of classic phase II detoxifying enzymes such as *GST*, *AOX*, and *UGT*, thereby conserving metabolic resources. Chl selectively activates the CncC (Nrf2) signaling pathway, remodels the redox state and detoxification network of *Drosophila*, thereby extending the lifespan of *Drosophila*. Research has concluded that there is no generalized age-related trend in activities of various antioxidant enzymes in *Drosophila* [69]. Nevertheless, the administration of dietary antioxidants should theoretically combat aging and/or age-related diseases and therefore extend lifespan [70]. However, KEGG analysis did not reveal enrichment of the CncC (Nrf2) pathway. This may be because there is no independent CncC pathway in KEGG, only a homologous mapping of the mammalian Nrf2 pathway, resulting in incomplete annotation.

The above experiments show that there are differences in varieties and gender between the results. Therefore, separate KEGG pathway enrichment analyses were conducted on differentially expressed genes in females and males. The results indicated that, besides the XM pathway, the lysosomal pathway was strongly associated with aging in females (Figure 6F). However, this pathway was inhibited, and we speculated that the reason for the downregulation of detoxification pathway may be the same as in the previous XM pathway, while the lifespan regulation pathway showed a significant association with aging in males (Figure 6G), with *InR’s* (insulin receptor homolog in flies) downregulation. This result may be related to the classical theory that dietary restriction can prolong life [48]. Caloric restriction is likely related to reduced GH/insulin/insulin-like growth factor-1 (IGF-1) levels, and InR (Insulin-like receptor), which is one of the IGF-1 signaling molecules [71].

Interestingly, results also show that the nutrient metabolism pathways of flies were affected after feeding Chl, such as pancreatic secretion pathway, protein digestion and absorption pathway, and starch and sucrose metabolism pathway in female flies, and lipid and atherosclerosis pathway. This is consistent with our previous experimental result (Section 3.5) that feeding Chl affects the nutrient metabolism and energy storage in flies. Thus, the effect of Chl on environmental stress and prolonging life may be related to these pathways.

### 3.7. Transcription Factor Motif Analysis

**Figure 7:**
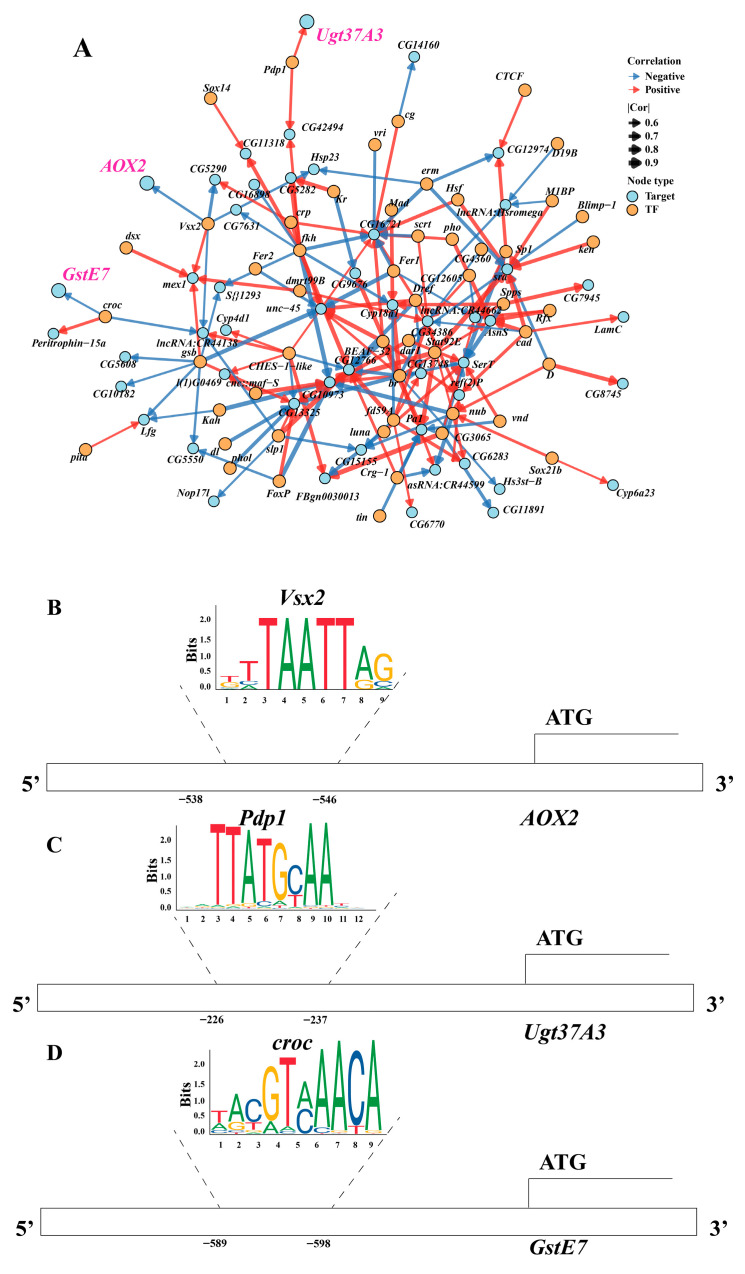

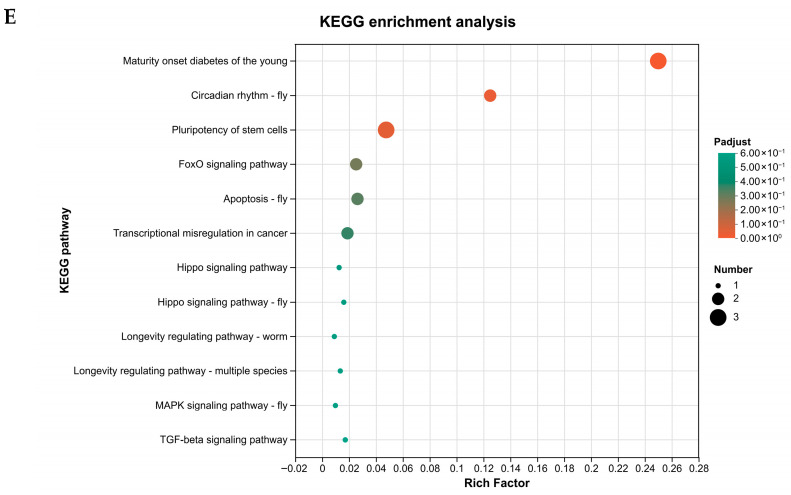
Transcription factor motif analysis of differentially expressed genes upon chlorophyll treatment. (**A**) Transcription factor regulatory map (screening within 1000 bp upstream of TSS, Cor > 0.5); (**B**–**D**) Transcription binding site maps (*Vsx2*, *Pdp1*, and *croc*); (**E**) TF (transcription factor) enrichment analysis.

Based on 118 differentially expressed transcription factor (TF) genes shared by both sexes annotated from RNA-seq, upstream regulatory factors were predicted for these TFs using RStudio (version 2026.01.2-418) combined with the Animal TFDB and JASPAR databases. The screening region was set from 1000 bp upstream of the transcription start site (TSS) to the TSS itself (0 bp), with a binding strength threshold of correlation (Cor) > 0.5. A transcription factor regulatory network was subsequently constructed (Figure 7A). As illustrated in the figure, three differentially expressed genes, namely *Aox2*, *Ugt37A3*, and *GstE7*, were selected as target genes to predict their upstream transcription factors. The results indicate that *Vsx2*, *Pdp1*, and *croc* regulated these three genes, respectively. Further analysis identified transcription factor binding motifs at the following positions: “GTTAATTAG” at 538–546 bp upstream of the *Aox2* TSS, “GCTTATGCAATT” at 226–237 bp upstream of the *Ugt37A3* TSS, and “AACGTCAACA” at 589–598 bp upstream of the *GstE7* TSS (Figure 7B–D). However, one GST gene (*GstD10*) was excluded from promoter analysis because its upstream 1000 bp sequence could not be reliably retrieved from the current genome annotation, possibly due to an incomplete gene model or its location near a scaffold boundary.

Subsequently, KEGG enrichment analysis was performed on these three transcription factors (*Vsx2*, *Pdp1*, and *croc*). The results (Figure 7E) show that they were enriched in signaling pathways, including FoxO, apoptosis, and longevity-regulating pathways. Although these pathways were not identical to those directly enriched by the differentially expressed genes in the transcriptome, they were functionally related, and both were closely associated with aging regulation [72,73]. In summary, the motif analysis supports the reliability of the transcriptomic results from a transcriptional regulatory perspective and provides initial insights into the potential upstream regulatory mechanisms through which chlorophyll may exert its effects.

## 4. Conclusions

This study demonstrates that Chl supplementation extends the mean lifespan of *Drosophila melanogaster*. Chl given at a certain dose (3.925 mg/L) prolonged longevity, increased athletic ability, and resistance to heat shock, cold shock, starvation stress, and oxidative stress in the flies. The prolongation of the flies’ lifespan is not related to restriction of dietary intake, sacrifice of reproductive capacity, or rhythm regulation. The change in the stress resistance of *Drosophila* may be caused by Chl’s effect on energy metabolism (glucose and triglyceride levels). This includes Chl’s enhancement of the activities of antioxidant enzymes (mainly CAT) and its inhibition of the production of MDA. Gene expression and qPCR studies display upregulated expression of *cat*, *Mrp4*, *Hsp68*, and downregulated expression of *GstD10* and *GstE7*, *Ugt37A3*, and *AOX2* in the XM pathway. The motif analysis supports the reliability of the transcriptomic results.

In summary, our findings demonstrate that Chl acts as a hormetic nutrient. Dietary supplementation with Chl optimizes energy substrates in *Drosophila* (reduces glucose and lipid levels and modulates nutrient metabolism pathways). In addition, it moderately and selectively activates the CncC (Nrf2) signaling pathway with upregulation of protective target genes (*cat*, *Mrp4*, *Hsp68*), decreases MDA content, increases CAT activity, and downregulates classic phase II detoxifying enzyme genes including *GstD10*, *GstE7*, *AOX2*, and *Ugt37A3*. Ultimately, Chl supplementation reshapes the detoxification network and converts this protective advantage at the cellular level into an observable survival advantage at the biological level, that is, significantly improves the resistance to oxidative stress, starvation, high temperature, low temperature, and other environmental stresses, extending the lifespan and motor activity of *Drosophila.* The benefits brought by Chl may be far more than changes in several indicators at the molecular level, but translated into the overall improvement of the organism’s “health resilience” and “viability”.

Nevertheless, the present study has several limitations that warrant further investigation. First, the optimal anti-aging dose of Chl remains to be determined, as the dose is a critical factor determining whether a compound exerts protective or toxic effects. Given that Chl shows a hormetic response in enhancing antioxidant capacity in *Drosophila*, additional dose–response studies are needed to identify the most effective concentration. In addition, the chlorophyll concentration used in this study was preliminarily determined through experiments in *Drosophila* as an in vivo model. Given interspecific differences in metabolism [74], the chlorophyll dosage requires further optimization, and its biological effects need to be verified in subsequent aging-related studies using mice and humans. Second, aging is a highly complex process, and the metabolism of dietary nutrients may differ across species. Since this study was performed only in the *Drosophila* model, the generalizability of our findings to other organisms, including mammals, remains to be validated. Finally, other potential mechanisms underlying the anti-aging effects of Chl remain unexplored. For example, whether the gut-brain-aging axis is involved in Chl-mediated longevity benefits requires further examination. Addressing these questions will be crucial for understanding the precise molecular mechanisms underlying Chl’s anti-aging effects and may uncover novel therapeutic targets for age-related diseases.

## Figures and Tables

**Table 1:** Formula of culture medium.

Yeast Medium		Grape Juice Medium		Medium for Hunger Experiment	
Ingredient	Per Liter Food	Ingredient	Per Liter Food	Ingredient	Per Liter Food
Water (mL)	1000	Water (mL)	800	Water (mL)	965
CaCl_2_ (g)	0.726	Sucrose (g)	15	Agar (g)	15
Sucrose (g)	31.62	Agar (g)	37.5	Ethyl Paraben (mL; 10% *w*/*v*)	30
Dextrose (g)	63.2	Grape juice (mL)	200	Propionic Acid (mL)	5
Cornmeal (g)	77.7	Ethyl Paraben (mL; 10% *w*/*v*)	15		
Yeast (g)	32.2				
Agar (g)	8				
Ethyl Paraben (mL; 10% *w*/*v*)	15				
Propionic Acid (mL)	5				

**Table 2:** List of primers used in gene expression study.

Gene Name	Primer Sequences (5′ to 3′)	Gene Name	Primer Sequences (5′ to 3′)
*AOX2*	F:TTCGGTGGAAACCTCTGTCG R:GCTCGAAGGAGTCCTCGATG	*GstE7*	F:ATTATTGAGGTCTATGACTTCCT R:GTCCACCTTTACGAAGACCT
*GstD10*	F:TGTGGAGTTCGATAAGAAGAC R:ATTGGCATACCGCTTGAAAT	*cat*	F:TGACTACAAAAACTCCCAAACG R:TTGATTCCAATGGGTGCTC
*sod1*	F:GGAAAACGCGCATAAACATT R:GGATTTCTGGATGTGGTGCT	*sod2*	F:AATTTCGCAAACTGCAAGC R:TGATGCAGCTCCATGATCTC
*Mrp4*	F:AAGAACGTGGACGGTCAAAC R:GCAAATTGTCGAACTCAGCA	*Hsp68*	F:TTAAGGATGGTGAGGCCAAG R:CACGGAATTGTTGGTGTTTG
*rp49*	F:GCACCAAGCACTTCATCC R:CGATCTCGCCGCAGTAAA		

**Table 3:** Life-span data for *Drosophila*.

Genetic	Groups	Flies (*n*)		Median Survival (d)		Mean Longevity (d ± SE)		Maximum Longevity (d ± SE)		Mean Increase (%)	
Background	Chl Level (mg/L)	Female	Male	Female	Male	Female	Male	Female	Male	Female	Male
Canton-S											
	LT (0)	159	185	48	30	45.74 ± 1.16	28.20 ± 0.70	70.20 ± 0.73	52.20 ± 2.04	−2.07	−8.41
	C (0)	163	163	51	30	46.71 ± 1.28	30.79 ± 0.78	70.20 ± 1.53	52.80 ± 1.53	—	—
	L (3.925)	197	198	57	36	52.32 ± 1.14	35.08 ± 0.60	74.40 ± 0.60	54.00 ± 1.34	12.01 ***	13.93 ****
	M (7.85)	196	167	57	33	50.57 ± 1.20	34.20 ± 0.73	73.80 ± 0.73	55.80 ± 1.53	8.26 *	11.07 **
	H (15.7)	197	198	54	33	48.78 ± 1.16	32.17 ± 0.68	71.40 ± 0.60	55.20 ± 0.73	4.43	4.48
W^1118^											
	LT (0)	226	252	57	51	52.70 ± 1.05	48.96 ± 0.77	82.80 ± 1.53	75.00 ± 1.20	−6.44 **	−2.90
	C (0)	249	229	60	54	56.33 ± 1.09	50.42 ± 0.85	84.60 ± 1.12	76.20 ± 1.53	—	—
	L (3.925)	198	238	63	57	56.52 ± 1.33	54.28 ± 0.94	86.40 ± 1.12	82.80 ± 0.73	0.33	7.66 ****
	M (7.85)	206	244	63	54	57.15 ± 1.34	51.30 ± 0.80	85.20 ± 0.73	81.00 ± 2.12	1.46	1.75
	H (15.7)	196	241	66	54	60.84 ± 1.31	50.63 ± 0.81	87.60 ± 1.12	76.20 ± 1.53	8.01 ***	0.42

* *p* < 0.05, ** *p* < 0.001, *** *p* < 0.001, **** *p* < 0.001 vs. control.

**Table 4:** Circadian rhythm data for *Drosophila*.

Genetic	Groups	Num	% Rhythmic	Tau	Power
Background	Chl Level (mg/L)				
Canton-S					
	0 (Light)	9	44.44	15.13 ± 3.13	49.63 ± 10.52
	0	10	40	16.9 ± 3.00	40.98 ± 5.52
	3.925	10	40	17.88 ± 3.39	47.2 ± 6.21
	7.85	11	36.36	23.63 ± 0.13	37.55 ± 6.61
	15.7	12	58.33 *	20.43 ± 2.18	42.77 ± 6.36
W^1118^					
	0 (Light)	16	100	23.84 ± 0.06	106.76 ± 8.23
	0	19	94.74	23.78 ± 0.06	100.58 ± 8.70
	3.925	15	100	23.87 ± 0.06	75.44 ± 8.08
	7.85	12	83.33 *	25.05 ± 0.65	75.85 ± 9.11
	15.7	17	82.35 **	23.79 ± 0.07	74.97 ± 6.69

* *p* < 0.05, ** *p* < 0.001 vs. control.

## Data Availability

The raw data supporting the conclusions of this article will be made available by the authors on request.
